# “Next generation sequencing identifies mutations in GNPTG gene as a cause of familial form of scleroderma-like disease”

**DOI:** 10.1186/s12969-017-0215-8

**Published:** 2017-12-29

**Authors:** Afolake T. Arowolo, Henry A. Adeola, Nonhlanhla P. Khumalo

**Affiliations:** Hair and Skin Research Laboratory, Division of Dermatology, Department of Medicine, Faculty of Health Sciences and Groote Schuur Hospital, University of Cape Town, Cape Town, South Africa

**Dear Editor**,

With great interest, we read Zrhidri et al.’s paper [1] which reports compound heterozygous mutations in exon 4 and 9 of the GNPTG gene, in a familial scleroderma-like disease. This novel finding represents an important addition to the family of genetic mutations previously associated with multisystemic fibrosis and scleroderma-like diseases in literature. Further, elucidating pathogenetic mechanisms of genetic systemic fibrosis could potentially lead to discovery of effective treatment of auto-immune systemic sclerosis and related diseases, and alleviate severe morbidity and mortality.

Although the authors focused on scleroderma-like manifestations found in Mucolipidosis type III (pseudo-Hurler polydystrophy), it would have also been useful to mention other genes associated with a scleroderma-like phenotype in their study discussion. Some examples are listed below:

- FAM 111B gene for scleroderma and multisystemic fibrosis-like hereditary fibrosing poikiloderma with tendon contractures, myopathy, and pulmonary fibrosis (POIKTMP) [2]
- RECQL4 gene for Rothmund Thomson Syndrome (RTS) [3],
- WRN gene for Werner syndrome (WS) [4]
- LMNA gene in Hutchinson-Gilford progeria syndrome (HGPS) [5]

Finally, considering the multifactorial etiology of fibrosis, it would be interesting to see how the new gene (GNPTG) compares to other genes involved in scleroderma-like diseases such as FAM 111B (POIKTMP), RECLQL4 (RTS), WRN (WS) and LMNA (HGPS); and if there are possible gene interactions, considering the similarities in the phenotype produced.

## References

[1] Zrhidri A, Amasdl S, Lyahyai J, Elouardi H, Chkirate B, Raymond L (2017). Next generation sequencing identifies mutations in GNPTG gene as a cause of familial form of scleroderma-like disease. Pediatr Rheumatol.

[2] Mercier S, Küry S, Shaboodien G, Houniet DT, Khumalo NP, Bou-Hanna C (2013). Mutations in FAM111B cause hereditary fibrosing poikiloderma with tendon contracture, myopathy, and pulmonary fibrosis. Am J Hum Genet.

[3] Larizza L, Roversi G, Volpi L (2010). Rothmund-thomson syndrome. Orphanet journal of rare diseases.

[4] Goto M, Okawa-Takatsuji M, Aotsuka S, Nakai H, Shimizu M, Goto H (2006). Significant elevation of IgG anti-WRN (RecQ3 RNA/DNA helicase) antibody in systemic sclerosis. Mod Rheumatol.

[5] Kim HK, Lee JY, Bae EJ, Oh PS, Park WI, Lee DS (2011). Hutchinson-Gilford progeria syndrome with G608G LMNA mutation. J Korean Med Sci.

